# New Methylcitrate Synthase Inhibitor Induces Proteolysis, Lipid Degradation and Pyruvate Excretion in *Paracoccidioides brasiliensis*

**DOI:** 10.3390/jof9010108

**Published:** 2023-01-13

**Authors:** Olivia Basso Rocha, Kleber Santiago Freitas e Silva, Thaynara Gonzaga Santos, Dayane Moraes, Leandro do Prado Assunção, Alexandre Melo Bailão, Célia Maria de Almeida Soares, Maristela Pereira

**Affiliations:** 1 Laboratory of Molecular Biology, Institute of Biological Sciences, Federal University of Goiás, Avenida Esperança, s/n, ICB2, Sala 206, Goiânia 74690-900, GO, Brazil; 2 Institute of Tropical Pathology and Public Health, Federal University of Goiás, Goiânia 74690-900, GO, Brazil

**Keywords:** antifungal, paracoccidiodomycosis, methylcitrate cycle, methylcitrate synthase

## Abstract

Background: Paracoccidioidomycosis is a systemic mycosis caused by the inhalation of conidia of the genus *Paracoccidioides*. During the infectious process, fungal cells use several carbon sources, leading to the production of propionyl-CoA. The latter is metabolized by the methylcitrate synthase, a key enzyme of the methylcitrate cycle. We identified an inhibitor compound (ZINC08964784) that showed antifungal activity against *P. brasiliensis*. Methods: This work aimed to understand the fungal metabolic response of *P. brasiliensis* cells exposed to ZINC08964784 through a proteomics approach. We used a glucose-free medium supplemented with propionate in order to simulate the environment found by the pathogen during the infection. We performed pyruvate dosage, proteolytic assay, dosage of intracellular lipids and quantification of reactive oxygen species in order to validate the proteomic results. Results: The proteomic analysis indicated that the fungal cells undergo a metabolic shift due to the inhibition of the methylcitrate cycle and the generation of reactive species. Proteolytic enzymes were induced, driving amino acids into degradation for energy production. In addition, glycolysis and the citric acid cycle were down-regulated while ß-oxidation was up-regulated. The accumulation of pyruvate and propionyl-CoA led the cells to a state of oxidative stress in the presence of ZINC08964784. Conclusions: The inhibition of methylcitrate synthase caused by the compound promoted a metabolic shift in *P. brasiliensis* damaging energy production and generating oxidative stress. Hence, the compound is a promising alternative for developing new strategies of therapies against paracoccidioidomycosis.

## 1. Introduction

Paracoccidioides spp. is the causative agent of Paracoccidioidomycosis (PCM), an endemic disease in Latin America. *Paracoccidioides brasiliensis* is the predominant species of the fungus in Brazil [1]. The disease causes skin lesions and mainly affects the lungs but it can reach several other organs and systems, including the nervous system. Infection occurs when individuals inhale fungal conidia that within the host undergo a thermodimorphic transition into the infective yeast-like stage [2].

The current PCM treatment is based on antifungals such as amphotericin and itraconazole but both may develop severe side effects [2]. Amphotericin targets membrane sterols and can lead to nephrotoxicity. Itraconazole inhibits CYP450 enzymes, causing side effects such as headaches and dizziness [3]. In addition, the PCM treatment is long, lasting up to 2 years. This fact might reduce the patient’s adherence to the treatment, leading to sequelae and mortality mainly in immunocompromised patients [4].

The success of the infection is related to the ability of the fungus to evade the immunological barriers and the harsh environment inside the host [5]. The availability of nutrients, such as glucose, is scarce within the host cells and pathogens regulate metabolic pathways, mainly those related to energy production in order to establish the infection. Thus, pathogen cells must produce energy from carbon sources other than glucose, such as fatty acids and amino acids. Alternatively, the fungus relies upon the activity of methylcitrate synthase (MCS), a key enzyme of the methylcitrate cycle, to metabolize propionyl-CoA. The latter is toxic and accumulates after fatty acid degradation [6,7,8].

The in silico search for new compounds for the treatment of fungal diseases is a strategy that has been widely used. Our research group has already identified antifungal compounds that target enzymes of the genus *Paracoccidioides* spp., such as isocitrate lyase (ICL) [9], malate synthase (MLS) [10] and MCS [11]. ZINC08964784 was the MCS inhibitor found by our study group in a virtual screening approach, this compound showed antifungal and fungicidal effects with an MIC value of 6.04 mM [11].

In this work, we aim to verify the proteomic response of *P. brasiliensis* yeast cells exposed to the MCS inhibitor ZINC08964784 in a glucose-free medium and supplemented with propionate, to simulate the environment found by the pathogen during the infection. We found that the fungus undergoes a metabolic shift due to the inhibition of methylcitrate cycle and the generation of reactive species. Proteolytic enzymes were induced, driving amino acids into degradation for energy production. In addition, glycolysis and the citric acid cycle were down-regulated while ß-oxidation was up-regulated. The accumulation of pyruvate and propionyl-CoA led the cells to a state of oxidative stress in the presence of ZINC08964784. Therefore, the compound is a promising alternative for developing new strategies of therapies against paracoccidioidomycosis.

## 2. Materials and Methods

### 2.1. Chemicals

The compound ZINC08964784 used in the experiments was acquired by the website Molport (USA). The chemical structure of the compound that identified ZINC08964784 as an MCS inhibitor was explored in our previous work. ZINC08964784 comprises multiple functional groups, such as amine, amide, ketone and ether groups. The nitrogen and oxygen of those groups contribute to the interaction of the compound with its target enzyme MCS [11].

### 2.2. Microorganism and Culture Conditions

*P. brasiliensis*—Pb 18 yeast cells were cultivated at 36 °C for 48 h under agitation in Fava–Netto liquid medium containing protease peptone, 1% peptone, 0.5% (*w*/*v*) meat extract, 0.5% (*w*/*v*) yeast extract, 4% glucose, 0.5% [NaCl], and pH 7.2. Posteriorly, 10^5^ cells were incubated with RPMI 1640 glucose-free medium supplemented with propionate 5 mM (Sigma-Aldrich, St. Louis, MO, USA) or in the presence of 6.04 µM of ZINC08964784, the concentration of the compound responsible for the fungicidal effects as identified in our previous work. 

The cell viability of *P. brasiliensis* yeast cells in the presence of the compound ZINC08964784 was analyzed through optical density measured at 600 nm wavelength and cells were counted via trypan blue staining in a Neubauer chamber. We monitored the time intervals of 6, 12, 24 and 48 h. The analysis was performed in triplicate and the statistical significance was assessed by the Student’s *t*-test where *p* ≤ 0.05 was considered significant.

### 2.3. Obtaining the Protein Extract

The cells were exposed to 6.04 µM of ZINC08964784 for 12 h in the treatment group (according to the results of the viability test), while in the control group they were cultured under the conditions mentioned above. Then, the cells were centrifuged at 3000× *g*, the supernatant was discarded and the cells were resuspended in ammonium bicarbonate buffer (57 mM, pH 8.8). The cells were subjected to mechanical lysis with the addition of glass beads and 5 cycles of 30 s in the bead beater disruptor (BioSpec, Bartlesville, OK, USA). Subsequently, they were centrifuged at 12,000× *g* and the supernatant with the protein extract was collected. The quality of the protein extracts was verified by SDS PAGE and quantified using the Bradford reagent (Sigma-Aldrich, St. Louis, MO, USA).

### 2.4. Protein Digestion and NanoUPLC-MSE Analysis

Sample aliquots (150 µg) composed by the equimolar mixture from each biological replica were prepared by tryptic enzymatic digestion. Initially, 75 µL of a 0.2% (*w*/*v*) RapiGEST™ SF (Waters, Milford, MA, USA) was added to the protein extract for 15 min at 80 °C, as a surfactant. Then, as a disulfide bridge-reducing agent, 2.5 µL of 100 mM dithiothreitol (DTT; GE Healthcare, Little Chalfont, UK) was added at 60 °C, for 30 min. This was followed by the addition of 300 mM iodoacetamine (GE Healthcare, Piscataway, NJ, USA), as an alkylating agent, which remained at rest for 30 min at room temperature and protected from the light. The digestion was performed by the addition of 30 μL of a 0.05 μg/μL trypsin solution (Promega, Madison, WI, USA) and incubated at 37 °C for 16 h. Subsequently, 30 µL of 5% trifluoroacetic acid (*v*/*v*) was added during 90 min at 37 °C for the surfactant precipitation. The samples were centrifuged at 13,000× *g*, for 30 min at 4 °C, until there was no precipitate, then the supernatant was lyophilized in a speed vacuum apparatus. Finally, the peptides were suspended in a solution containing 20 mM of ammonium formate and 200 fmol/μL of PHB (MassPREP™ Digestion Standard). 

Samples were analyzed by nanoscale liquid chromatography (LC, nanoACQUITYTM UPLC, Waters Corporation, Manchester, UK) coupled with tandem mass spectrometry (MSE) according to [11]. Nanoscale liquid chromatography was carried out using an ACQUITY UPLC^®^ M-Class System (Waters Corporation, Milford, MA, USA). The peptides were separated on a system equipped with a first-dimension pre-column (ACQUITY PLUS UPLC M-Class HSS T3 1.8 μm 75 μm × 150 mm). To perform the second dimension, fractions were trapped onto a trapping column (ACQUITY UPLC M-Class Symmetry C18 Trap Column, 100A, 5 μm, 2D, V/M, 180 μm × 20 mm), in line with the analytical column (ACQUITY PLUS UPLC M-Class HSS T3 1.8 μm 75 μm × 150 mm). The peptides were subject to separation using a gradient 11.4, 14.7, 16%, 20.7 and 50% acetonitrile condition, in a flow of 2000 µL/min.

The mass spectra were obtained in a Synapt G1 HDMS™ mass spectrometer (Waters Micromass, Manchester, UK), equipped with a positive ionization nanoelectrospray (nanoESI+) and a quadrupole/time-of-flight analyzers (Q-TOF), ready to operate in TOF V-mode in an argon suspension collision chamber. [GLU1]-Fibrinopeptide B (GFB) were used as a mass-to-charge (*m*/*z* 785.8426, Sigma Aldrich, St. Louis, MO, USA) for calibration during the sample analysis. 

The mass spectra data were processed in Protein Lynx Global Server version 2.4 (PGLS, Waters Corporation, Manchester, UK) using the *P. brasiliensis* (Pb18) database (https://www.uniprot.org/proteomes/UP000001628; accessed on 25 November 2021) in forward and reverse directions. Proteins were considered regulated with at least a 1.5-fold change difference between conditions.

### 2.5. Pyruvate Dosage 

The amount of pyruvate in the medium was measured using the pyruvate assay kit (Sigma Aldrich, St. Louis, MO, USA). Cells were cultured for 12 h in the presence of the compound, while control cells were cultured solely in the medium. Then, 1 mL of medium was retrieved from the samples and centrifuged at 2000× *g* and the supernatant was collected. Dosage was performed according to the manufacturer’s instructions. The readings were performed in triplicate for each condition and the statistical difference was verified with the Student’s *t*-test. Values of *p* ≤ 0.05 were statistically significant.

### 2.6. Azocasein Assay

The azocasein assay was performed in order to verify the proteolytic activity of enzymes [12]. The cells were cultured for 12 h in the presence of ZINC08964784 and without the compound in the control samples. Proteins were extracted and 150 µg of total protein extract was used for the assay. Azocasein was diluted to 5 mg/mL in buffer containing 25 mM Tris-HCl, 200 mM NaCl, 25 mM CaCl 2, 0.05% (*v*/*v*) Nonidet P-40 and 0.01% (*w*/*v*) NaN_3_. Positive control was based on well-known protease inhibitors (1 mM of PMSF, a serine protease inhibitor and 5 mM of EDTA, a metalloprotease inhibitor). Azocasein assays with significant differences were determined by statistical analysis using the Student’s t-test and values of *p* ≤ 0.05 were statistically significant.

### 2.7. Dosage of Intracellular Lipids

The quantification of the intracellular lipid content was performed by staining yeast cells exposed to the compound for 12 h and the control samples with fluorescent dye Nile Red (Sigma-Aldrich, St. Louis, MO, USA). The samples were stained with 1 µg/mL of Nile Red (2000 µg/mL) for 15 min at room temperature protected from light. The cells were centrifuged and washed with PBS two times and analyzed in a fluorescence microscope (Zeiss Axiocam, MRc-Scope A1) using a wavelength of 515–575 nm. The pixel intensity was quantified in the AxioVision software (Carl Zeiss, Oberkochen, Germany). Cells that emitted fluorescence, with delimited borders and no overlapping areas were considered for the analyses. The samples were analyzed in triplicate. Statistical difference between treated and control samples was determined using Student’s *t*-test and values of *p* ≤ 0.05 were statistically significant.

### 2.8. Quantification of Reactive Oxygen Species

The quantification of reactive oxygen species (ROS) was performed using dichlorodihydrofluorescein 2′, 7′-diacetate dye (DCFH-DC) (Sigma-Aldrich, St. Louis, MO, USA). Initially, 1 × 10^5^ cells/mL were incubated in the presence of 6.04 µM of ZINC08964784. Cells grown in RPMI 1640 medium without the compound were used as the control. Aliquots of 1 mL were collected after 12 h and 25 μM of DCFH-DC was added. The mixture was kept protected from light for 30 min. The cells were washed twice with PBS 1x and observed under a fluorescence microscopy (Zeiss Axiocam MRc—Scope A1) using λex equal to 490 nm and λem equals to 516 nm. The quantification of cells based on the fluorescence intensity was performed using the AxioVision V 4.8.2.0 (Carl Zeiss). Pixels density and the area of cells were measured. The statistical difference between the control and treated samples was assessed using Student’s t-test and values of *p* ≤ 0.05 were statistically significant.

## 3. Results

### 3.1. Proteomic Profile of P. brasiliensis after Treatment with ZINC08964784

**Figure 1:**
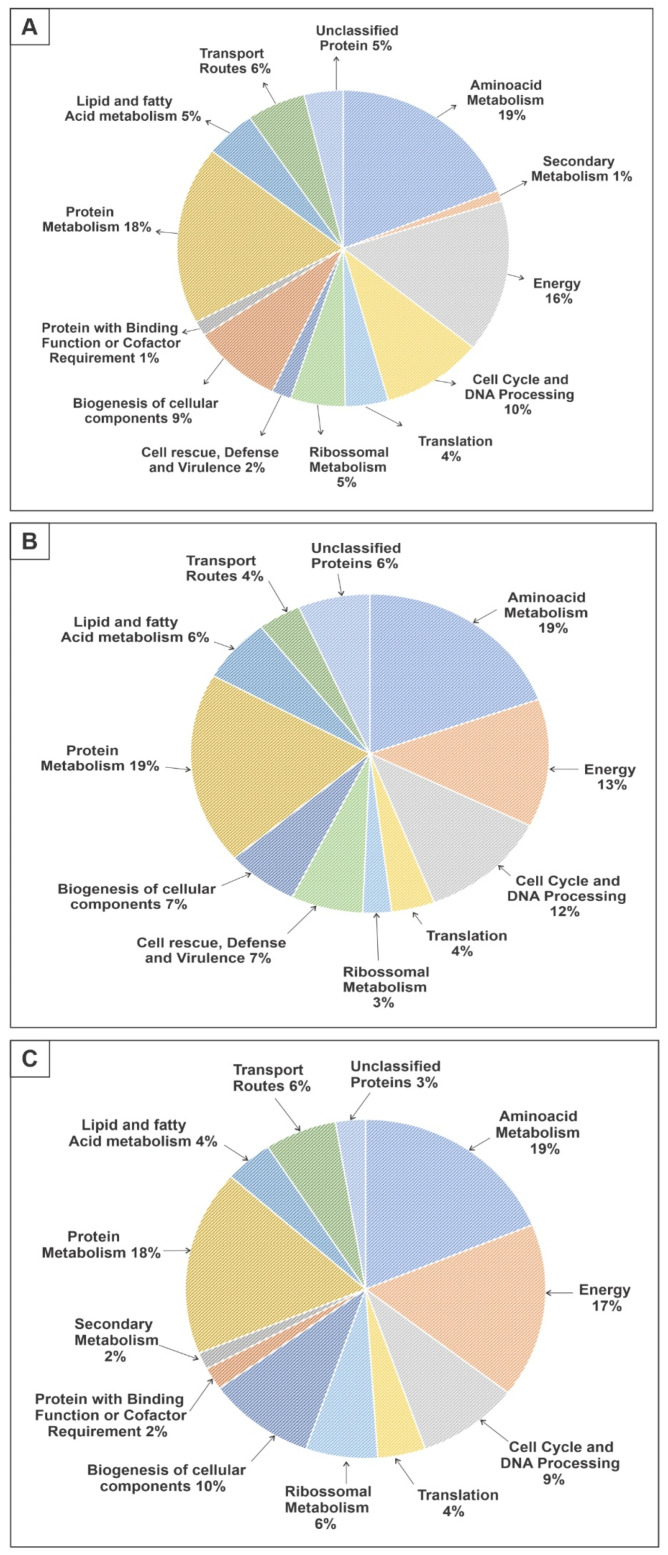
Functional categories of proteins that were regulated after ZINC08964784 treatment of *P. brasiliensis* yeast cells. (**A**) Pie chart showing the functional categorization of all of the 263 proteins that were differently expressed. (**B**) Pie chart highlighting the functional categorization of 77 proteins with increased expression after ZINC08964784 treatment. (**C**) Pie chart highlighting the functional categorization of 186 proteins that had reduced expression after ZINC08964784 treatment.

Protein extracts from *P. brasiliensis* samples treated with the compound ZINC08964784 were analyzed by nanoUPLC-MS^E^ with the aim of identifying differentially expressed proteins. This assay identified 263 differentially expressed proteins. Among those, 77 proteins were up-regulated (Supplementary Table S1), while 186 were down-regulated (Supplementary Table S2) after treatment with the compound ZINC08964784. A 1.5-fold change was used as a threshold in order to determine up- and down-regulated proteins. More than 96% of the total identified proteins were classified into biological categories, while only 4% of them were unclassified proteins and could not be categorized using the Uniprot system (Figure 1A).

The up-regulated proteins were classified into 10 different groups. More than 60% of those proteins were related to categories such as amino acid metabolism (19%), protein metabolism (19%), energy production (13%) and cell cycle and DNA processing (12%). Additionally, biogenesis of cellular components (7%), cell rescue, defense and virulence (7%), lipid and fatty acid metabolism (8%), transport routes (4%), translation (4%) and ribosomal metabolism (3%) were also up-regulated (Figure 1B).

The down-regulated proteins were classified into 12 different groups. More than 60% of those proteins were related to categories such as amino acid metabolism (19%), protein metabolism (18%), energy production (17%) and biogenesis of cellular components (10%). Additionally, cell cycle and DNA processing (9%), transport routes (6%), ribosomal metabolism (6%), translation (4%), lipid and fatty acid metabolism (4%), protein with binding function or cofactor requirement (2%) and secondary metabolism (2%) were also down-regulated (Figure 1C).

### 3.2. Exposure to MCS Inhibitor Leads to Pyruvate Excretion

**Figure 2:**
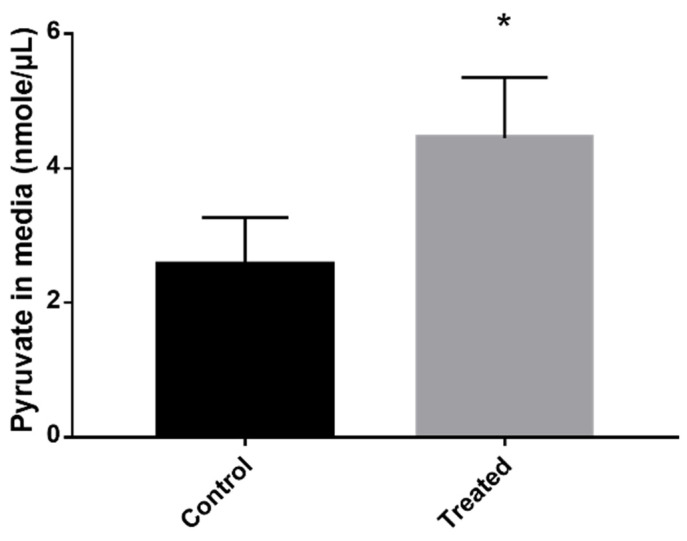
Exposure to the MCS inhibitor increased pyruvate excretion. The cells were exposed or not exposed to ZINC08964784 for 12 h and the concentration of pyruvate in the medium was assessed and compared. The statistically significant difference between the control and treated samples was verified using Student’s *t*-test. The * means significant difference (*p* ≤ 0.05) between samples.

Inhibition of the methylcitrate cycle leads to the accumulation of intracellular pyruvate that consequently is secreted into the extracellular environment [12]. Thus, we verified whether the amount of pyruvate in the culture medium of cells treated with the inhibitor was greater than the control cells. The quantification showed that after exposure to the compound, the cells secreted higher levels of pyruvate to the extracellular environment than the control samples (Figure 2).

### 3.3. Inhibition of MCS Increases Fungal Proteolytic Activity

**Figure 3:**
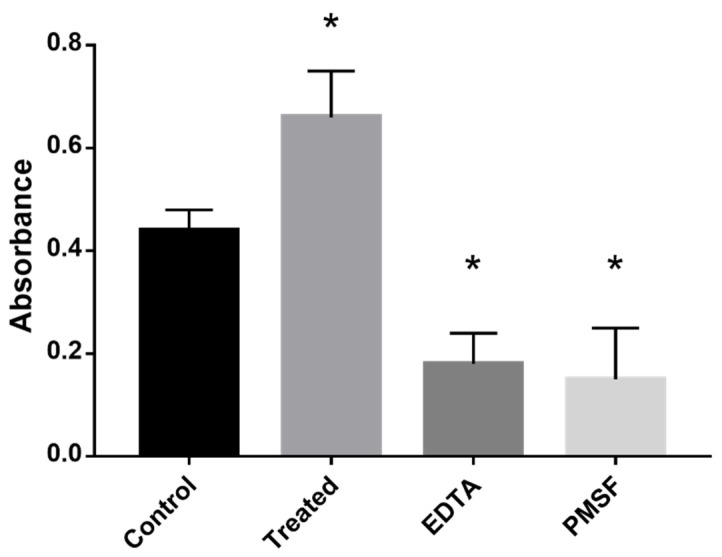
*P. brasiliensis* cells exposed to the MCS inhibitor showed increased proteolytic activity. The cells were exposed for 12 h to the compound in the experimental group. Negative controls comprised samples without ZINC08964784 and positive controls comprised PMSF, a serine protease inhibitor and 5 mM EDTA, a metalloprotease inhibitor. The quantification of the proteolytic activity was performed through the azocasein assay. The statistically significant difference between the control and treated samples was verified using Student’s *t*-test. The * means significant difference (*p* ≤ 0.05) between samples.

The absence of glucose and supplementation with propionate increases the oxidative stress in the fungal cells. This contribute to the inhibition of glycolysis and activation of methylcitrate cycle to metabolize propionyl-CoA [13]. The addition of the MCS-inhibiting compound disrupt the methyl–citrate cycle, leading the microorganism to regulate alternative pathways to produce energy. The breakdown of proteins to produce amino acids that could supply energy led to the up-regulation of enzymes such as cysteine dioxygenase (CD) and the glycine cleavage system (GCS) (Supplementary Table S1). We found that treatment of cells with the compound increased proteolysis in the samples treated with ZINC08964784 when compared to the control samples (Figure 3).

### 3.4. Exposure to MCS Inhibitor Increases Intracellular Lipid Degradation

**Figure 4:**
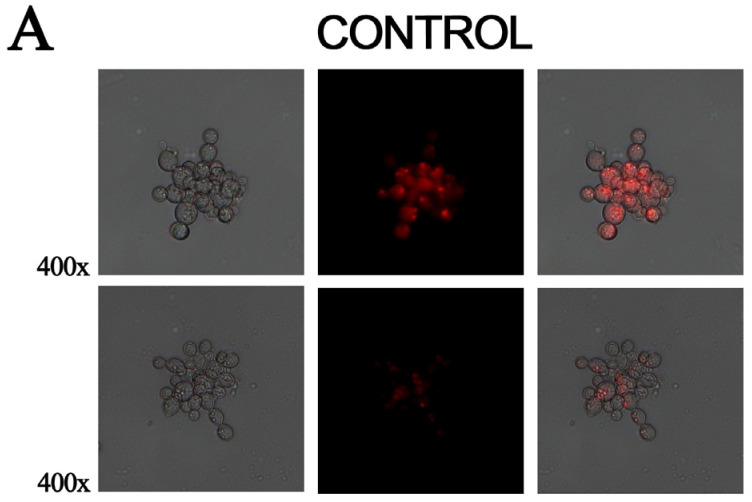

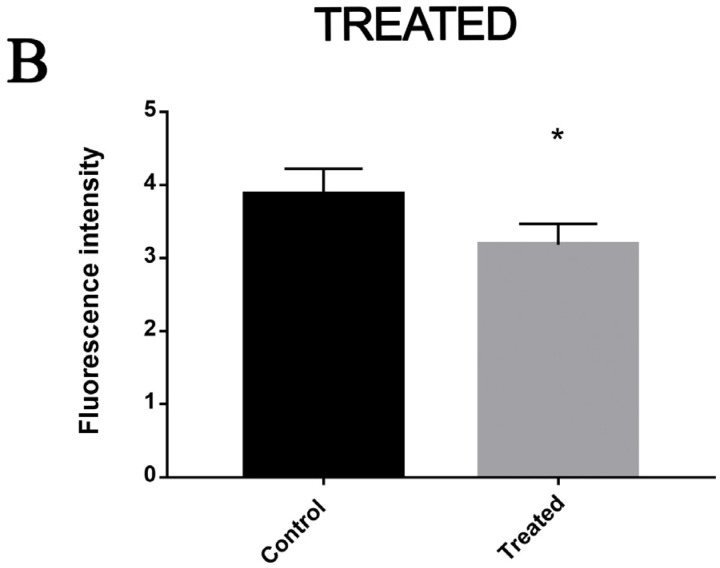
The inhibition of MCS by the compound ZINC08964784 increases the degradation of fatty acids. (**A**) Fluorescence photomicroscopy stained with Nile red showing lipid deposits. Image obtained at 400× magnification. (**B**) Quantification of lipid accumulation. The fluorescence intensity (in pixels) of the stained cells was measured using AxioVision software (Carl Zeiss, Oberkochen, Germany). Error bars represent the standard deviation of three biological replicates. The statistically significant difference between the control and treated samples was verified using Student’s *t*-test. The * means significant difference (*p* ≤ 0.05) between samples.

Cells that were exposed to the MCS inhibitor for 12 h overexpressed β-oxidation enzymes, such as enoyl-COA hydratase and 3-ketoacyl-COA thiolase. Staining samples with Nile red demonstrated that the lipid deposits were reduced in cells treated with the compound and consequently showed reduced fluorescence compared to the control samples. This indicates that lipids are metabolized in a higher level in treated samples (Figure 4).

### 3.5. Exposure to MCS Inhibitor Causes Oxidative Stress 

**Figure 5:**
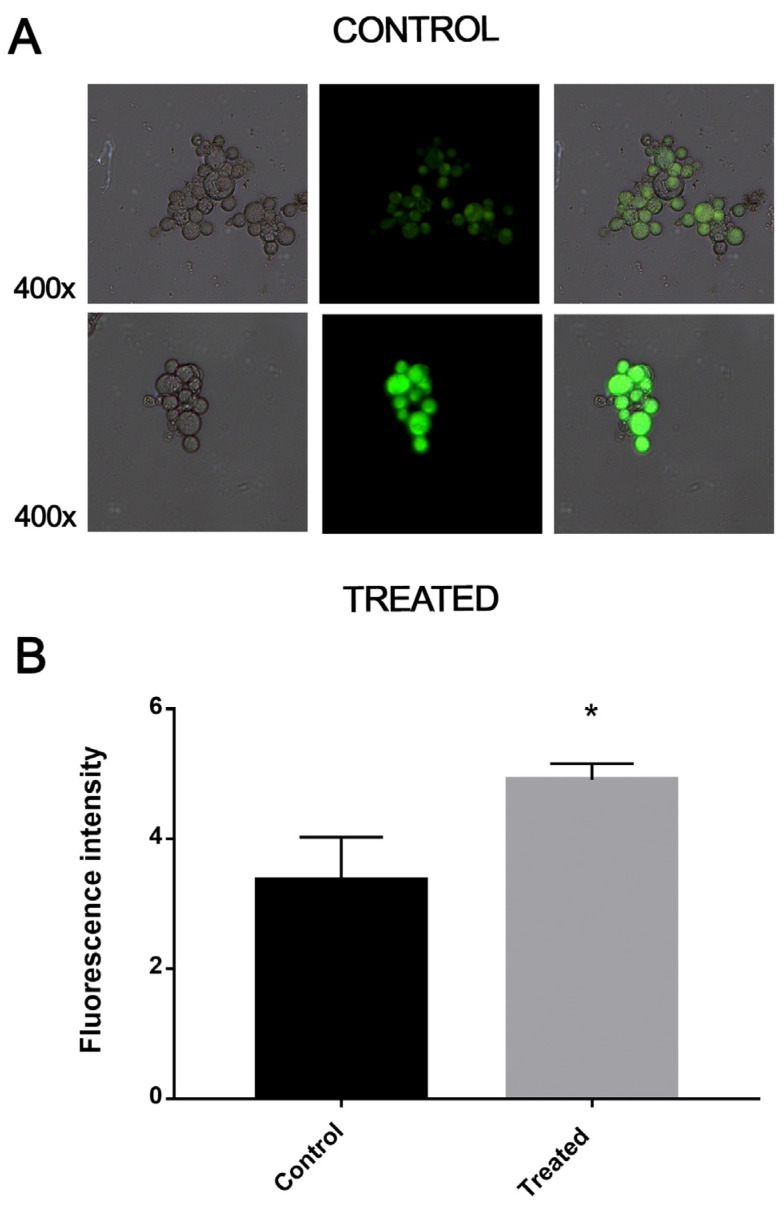
After exposure to ZINC08964784, *P. brasiliensis* cells showed increased levels of oxidative stress. (**A**) Fluorescence photomicroscopy of cells stained with DCFH-DC that labels reactive oxygen species. Image obtained at 400× magnification. (**B**) Quantification of lipid accumulation. The fluorescence intensity (in pixels) of the stained cells was measured using AxioVision software (Carl Zeiss, Oberkochen, Germany). Error bars represent the standard deviation of three biological replicates. The statistically significant difference between the control and treated samples was verified using the Student’s *t*-test. The * means significant difference (*p* ≤ 0.05) between samples.

Here, enzymes related to the oxidative stress response were found to be up-regulated, such as superoxide dismutase and thioredoxin. We verified whether the MCS-inhibiting compound would cause oxidative stress in fungal cells. The exposure to the inhibitor increased the production of ROS in samples treated with ZINC08964784 compared to the control group. This indicates that exposure to the compound led to oxidative stress in cells (Figure 5).

## 4. Discussion

Systemic fungal diseases are prevalent worldwide. The PCM treatment is based on antifungals such as sulfonamide, azole derivatives and amphotericin B [14] and requires a relatively long time. The cytotoxicity of those drugs enables the development of side effects and eventually the abandonment of the treatment [2]. Hence, new antifungal compounds could contribute to a better prognosis. Prototype compounds with antifungal features have been extensively investigated by our research group in order to propose more efficient therapeutic approaches against PCM [15]. 

Virtual screening and molecular docking assay along with experimental approaches resulted in the determination of antifungal compounds against specific targets of *P. brasiliensis*. The main targets are isocitrate lyase [9], MCS [11] and homoserine desidrogenase [16]. The latter was selected as an MCS prototype inhibitor and we chose this compound in order to establish the proteomic profile of *P. brasiliensis* cells in the presence of ZINC08964784. Considering these results and its ability to inhibit MCS, the ZINC08964784 compound is worth being investigated as an antifungal prototype.

**Figure 6:**
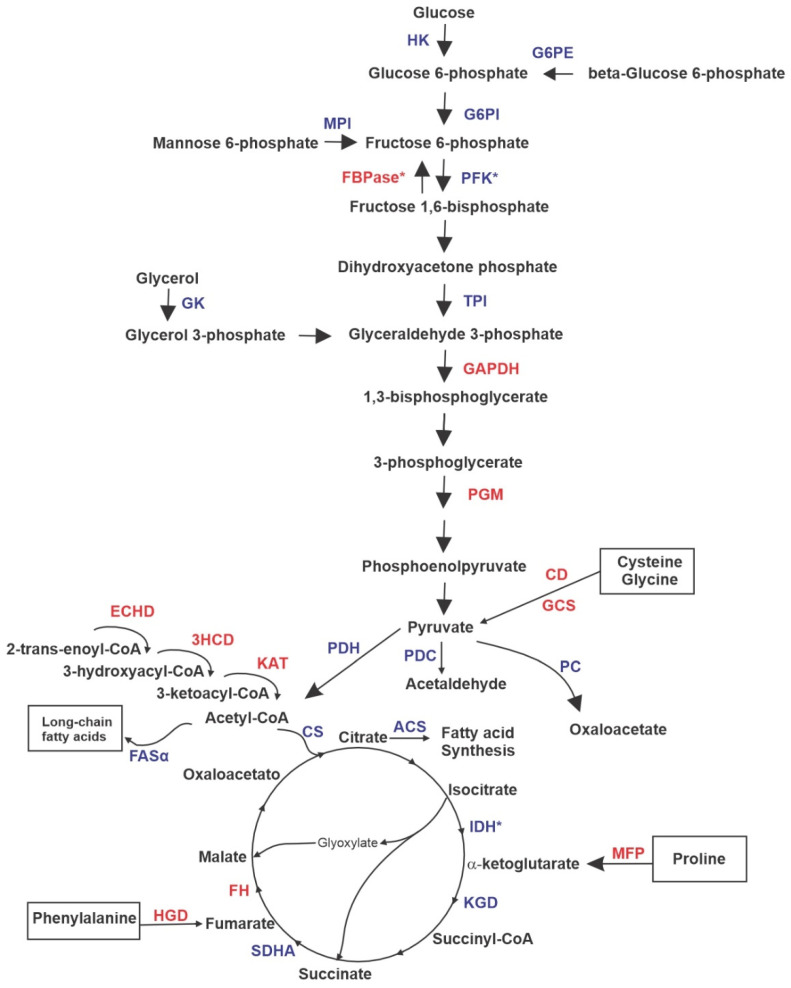
Model depicting metabolic changes after *P. brasiliensis* yeast cells were treated with ZINC08964784. The figure highlights proteins that are regulated in metabolic pathways (glycolysis, TCA, β-oxidation, fatty acid biosynthesis and amino acid degradation). PFK—phosphofructokinase; FBPase—fructose-bisphosphatase; HK—hexokinase; G6PE—glucose-6-phosphate 1-epimerase; MPI—mannose-6-phosphate isomerase; TPI—triose phosphate isomerase; GAPDH—glyceraldehyde-3-phophate dehydrogenase; GM—phosphoglycerate mutase; GK—glycerol kinase; CD—Cysteine dioxygenase; GCS—glycine cleavage system; PDH—pyruvate dehydrogenase; PDC—pyruvate decarboxylase; PC—pyruvate carboxylase; MFP—multifunctional fusion protein; HGD—homogentisate 1,2-dioxygenase; CS—citrate synthase; IDH—isocitrate dehydrogenase; ACS—acetyl-CoA synthetase; FASα—fatty acid synthetase alpha; KGD—alpha-ketoglutarate dehydrogenase; SDHA—succinate dehydrogenase complex flavoprotein subunit A; FH—fumarate hydratase; ECHD—enoyl-CoA hydratase; 3HCD—3-hydroxybutyryl-CoA dehydrogenase; KAT—3-ketoacyl-CoA thiolase. Down-regulated proteins are blue and up-regulated proteins are red. The asterisks indicate enzymes that are points of regulation within the pathways described.

The analysis of differentially expressed proteins and validation assays allowed us to point to the main metabolic processes altered by the presence of ZINC08964784. The most important alterations promoted by the presence of the compound in *P. brasiliensis* yeast cells are discussed in this section. Clearly, glycolysis is down-regulated while gluconeogenesis is up-regulated. This is indicated by the reduced expression of phosphofructokinase (PFK), which is a glycolysis regulation point and the increased expression of fructose-bisphosphatase (FBPase), which is a gluconeogenesis regulation point (Figure 6). This scenario represents cells that grow under glucose starvation, such as the micro-environment found within macrophages. In addition to glucose starvation, the methyl–citrate cycle inhibition, the consequent accumulation of propionate and cytotoxicity of the compound may be causing the reduced fungal cell growth [12,17,18]. Similarly, *Histoplasma capsulatum* is able to survive within macrophages, and grow and express several virulence factors if gluconeogenesis is active. This shift to gluconeogenesis is induced by the lack of available glucose inside macrophages [19]. Other fungal species have the ability to induce gluconeogenesis when glucose is not available, such as *Aspergillus nidulans* that grow on gluconeogenic carbon substrates, glutamate and ethanol [20] and *Yarrowia lipolytica* that grows on ethanol as a sole carbon source [21]. Interestingly, this is coherent with the fact that the fungal cells use alternative sources of energy, survivability requires the production of carbohydrates and MCS inhibition may intensify this phenomenon. Pyruvate and acetyl-CoA accumulation due to up-regulation of beta-oxidation and TCA down-regulation may contribute to the glycolysis inhibition [12].

The metabolism of pyruvate is clear to be grasped according to the proteomic results. Pathways that release pyruvate are up-regulated and those that consume pyruvate are down-regulated (Figure 6). Interestingly, this fact is supported by the knowledge that enzymes that metabolize acetyl-CoA are inhibited by propionyl-CoA via negative feedback [12]. CD takes part in the cysteine degradation that yields pyruvate [22] and GCS takes part in the degradation of the amino acids glycine and serine, yielding pyruvate as well [23]. Here, the destination of pyruvate is down-regulated since enzymes that could feed pathways that depend on pyruvate are with reduced expression. Pyruvate dehydrogenase (PDH) converts pyruvate into acetyl-CoA in order to feed TCA, pyruvate decarboxylase (PDC) converts pyruvate into acetaldehyde acting as a virulence factor and pyruvate carboxylase (PC) catalyzes the formation of oxaloacetate, which could be used to feed TCA, gluconeogenesis or even other pathways. Interestingly, all of those three enzymes are down-regulated. It has been shown that pyruvate accumulation is the first line of cell defense against several types of stress in a fungal cell [24]. Clearly, the presence of the MCS inhibitor causes a considerable amount of stress to the pathogen cells under treatment.

Other enzymes that can provide intermediaries to feed TCA are up-regulated, such as multifunctional fusion protein (MFP) and homogentisate 1,2-dioxygenase (HGD). These enzymes are related to the biosynthesis of α-ketoglutarate and fumarate, respectively, and they both can be driven either to TCA or gluconeogenesis [25,26]. The latter is up-regulated in treated cells. In addition, isocitrate dehydrogenase (IDH), which is a point of regulation of TCA, is down-regulated in the presence of ZINC08964784. Eventually, *P. brasiliensis* cells under treatment with the compound induce the β-oxidation pathway, and the result of this scenario is the release of acetyl-CoA, which could be used to feed TCA, but citrate synthase (CS) is down-regulated (Figure 6). ACS (acetyl-CoA synthetase) and FASα (fatty acid synthetase alpha) are also down-regulated, the former converts citrate into fatty acids and the latter promotes the synthesis of long-chain fatty acids. The β-oxidation pathway is also induced in fungal species during infection, such as *Cryptococcus neoformans* [27] and *Candida albicans* [28] and this induction responds to the accumulation of propionyl-CoA as well [12].

We verified that the MCS inhibition causes an excretion of pyruvate into the medium. This is due to the accumulation of propionyl-CoA, which leads to the non-oxidation of the pyruvate that accumulates in the intracellular medium until it is secreted by the cells. This same condition was found in *Aspergillus nidulans* mutant strains that had the MCS deleted and that were grown on propionate. The studies demonstrated an increase in the secretion of pyruvate of these strains and high concentrations of intracellular proponil-CoA that was responsible for inhibiting the activity of PC causing the accumulation of pyruvate, which was followed by its excretion [12]. In *Aspergillus fumigatus* that had the MCS gene deleted, an increase in pyruvate secretion was also found according to the amount of propionate in the medium [29].

Enzymes with proteolytic activities were found to be up-regulated after exposure to ZINCO8964784, such as the proteasome complex, this event may be occurring to generate amino acids for energy production, since glucose is not available and the methylcitrate cycle is inhibited. Several homeostatic roles of proteases have already been described in fungi, including in the genus *Paracoccidioides* spp., such as the cleavage of nutrients and the relationship between the excretion of these proteins and pathogenicity [13]. The increase in proteolytic activity may also be related to the attempt to degrade nutrients in the medium, as it happens during infection, as already demonstrated in *P. brasiliensis*, the activation of proteolytic activity during growth in different culture media [30].

Another form of energy production is the degradation of fatty acids; such an event was confirmed in our study by the induction of fatty acid oxidation and decreased content of lipid deposits in cells treated with MCS inhibitor. The β-oxidation pathway produces acetyl-CoA and propionyl-CoA; the latter would enter the methylcitrate cycle to be converted into pyruvate, as demonstrated in *A. nidulans* [31]. In *Mycobacterium tuberculosis*, the genes related to the degradation of fatty acids are also overexpressed during the infection of mice, where the environment is hostile and with a shortage of nutrients [32].

In our proteomic analysis, four antioxidant enzymes (three SOD isoforms and thioredoxin) were positively regulated, indicating that exposure to ZINCO8964784 increases the production of ROS. The overexpression of such enzymes reflects a response against the effects caused by high levels of ROS. These same enzymes are overexpressed in the stress response of *C. albicans* during the host colonization process [33]. In *H. capsulatum* during infection that leads to increased oxidative stress, it was shown that the fungus increases the expression of transcripts related to protein degradation and detoxification [34], as was demonstrated by our proteomic profile of *P. brasiliensis* exposed to this MCS inhibitor compound.

## 5. Conclusions

The search for a new treatment against PCM is still challenging. Finding more effective compounds and with less side effects are the key points. In this way, compounds that inhibit essential and exclusive enzymes of the fungus are promising. We demonstrated that the MCS inhibitor ZINC08964784 is a potential candidate for alternative treatment against the disease. The compound showed fungicidal activity and several metabolic alterations in the proteomic profile of *P. brasiliensis*. The presence of the compound inhibited the methyl–citrate cycle and stimulated propionyl-CoA accumulation, culminating with glycolysis and TCA repression, degradation of amino acids and beta oxidation and gluconeogenesis up-regulation. Therefore, ZINC08964784 is a promising alternative for developing new strategies of therapies against paracoccidioidomycosis.

## Data Availability

The data presented in this study are available on request from the corresponding author. The data are not publicly available because it is partially related to a subsequent publication.

## Supplementary Materials

The following supporting information can be downloaded at: https://www.mdpi.com/article/10.3390/jof9010108/s1. Table S1: Proteins up-regulated by *P. brasiliensis* with increase in abundance after exposure to ZINC08964784. Table S2: Proteins down-regulated by *P. brasiliensis* with increase in abundance after exposure to ZINC08964784.
