# Supplementary material for: New Methylcitrate Synthase Inhibitor Induces Proteolysis, Lipid Degradation and Pyruvate Excretion in Paracoccidioides brasiliensis
Source: J Fungi (Basel). 2023 Jan 13;9(1):108. doi: 10.3390/jof9010108 (PMC9865517; doi:10.3390/jof9010108)
Supplement: Supplementary file 1 [file jof-09-00108-s001.zip › jof-2142576-supplementary.pdf]

Table S1. ***P. brasilensis*** **proteins up-regulated after exposure to ZINCO8964784.**

Acession number	Protein description	Fold change
	**1. Metabolism**	
	**1.1 Amino acid metabolism**	
PADG_04689	Arginine-requiring protein 2	*
PADG_01305	Arginosuccinase	*
PADG_08406	O-acetylhomoserine (Thiol)-lyase	1,59115327034609
PADG_03522	S-methyl-5'-thioadenosine phosphorylase	2,32667694163372
PADG_02726	Cysteine synthase	2,9376962693129
PADG_01418	Cysteine dioxygenase	4,04033613445378
PADG_02914	Glycine cleavage system	2,94730228383365
PADG_05085	1-pyrroline-5-carboxylate dehydrogenase	2,45474443678813
PADG_08466	Homogentisate 1,2-dioxygenase	1,86643065078536
PADG_08265	Methionine aminopeptidase 2	1,849525358
PADG_07460	Vacuolar aminopeptidase	1,88366292535186
PADG_04167	Aspartyl aminopeptidase	1,61837358773873
PADG_03595	Xaa-Pro aminopeptidase P	1,75478054715039
PADG_06314	Carboxypeptidase Y homolog A	2,75105442960618
PADG_01963	Glycine cleavage system H protein	*
PADG_06490	Formamidase	2,94183014446193
	**1.2 Lipid, fatty acid and isoprenoid metabolism**	
PADG_03176	Phosphatidylglycerol/phosphatidylinositol transfer	1,62049291803496
PADG_01209	enoyl-CoA hydratase	1,67712136842871
PADG_01687	3-ketoacyl-CoA thiolase	1,74358133335194
PADG_01228	3-hydroxybutyryl-CoA dehydrogenase	2,49435486248268
PADG_01233	4HBT domain-containing protein	2,58440409683426
	**1.3 C- compound metabolism**	
PADG_06202	NodB homology domain-containing protein	3,13080495356037
PADG_01265	Mesaconyl-C4 CoA hydratase	4,83296880395994
PADG_05063	α-1,3/1,6-mannosyltransferase ALG2	*
PADG_08402	27 kDa antigen	4,18535639725619
	**2. Energy**	
	**2. 1 Glycolysis and gluconeogenesis**	
PADG_05109	Phosphoglycerate mutase	1,56001373469154
PADG_01706	Fructose-bisphosphatase	2,34766742463141
PADG_02411	Glyceraldehyde-3-phosphate dehydrogenase	2,75158074688034
	**2.2 Pentose-phosphate pathway**	
PADG_04989	D-ribose-5-phosphate ketol-isomerase	4,71979277769313
	**2.3 Tricarboxylic-acid pathway**	
PADG_08119	Fumarate hydratase	1,59699059226
PADG_04710	Citrate synthase	1,84448668519681
PADG_06494	Dihydrolipoyl dehydrogenase	2,11324750806761
PADG_08013	Succinate dehydrogenase iron-sulfur subunit	*
	**2.4 Electron transport chain**	
PADG_06600	Cytochrome b5 heme-binding	*
PADG_06978	Cytochrome c	*

	**3. Protein metabolism**	
PADG_07190	20S proteasome subunit alpha 2	1,5626828857261
PADG_08442	Proteasome subunit alpha type	1,70018643868519
PADG_04587	Glutathione peroxidase	1,70788200046094
PADG_08087	Proteasome subunit beta	1,77001221853727
PADG_03727	Proteasome endopeptidase complex	10,3546406644998
PADG_03967	proteasome core particle subunit beta 6	2,10382198476063
PADG_06546	Puromycin-sensitive aminopeptidase	2,57304933362639
PADG_06290	Proteasome α1 domain-containing protein	2,65879125066687
PADG_01605	Polyubiquitin	20,3029191423405
PADG_01605	Polyubiquitin	20,3029191423405
PADG_07422	Serine proteinase	3,56822851493102
PADG_08369	Hsp60	2,68691506372572
PADG_04062	Hsp90	8,08787148594377
PADG_08587	Peptidylprolyl isomerase	3,19823008849557
	**4. Cell rescue, defense and virulence**	
PADG_07418	Superoxide dismutase Cu-Zn	2,19821988216121
PADG_01755	Superoxide dismutase Cu-Zn	1,66673379912934
PADG_01954	Superoxide dismutase, divalent metal cation	5,02616449606946
PADG_02764	Thioredoxin	1,74549592870424
PADG_04636	DLH domain-containing protein	1,58735339039221
PADG_01857	DLH domain-containing protein	7,3285015060241
	**5. Translation**	
PADG_03440	Prolyl-tRNA synthetase	2,74634942709436
PADG_01387	60S ribosomal protein L7	3,35629829624802
PADG_05883	60S ribosomal protein L25	1,85129358830146
	**6. Biogenesis of cellular components**	
PADG_00995	Ubiquitin-40S ribosomal protein S27a	3,89706544243406
PADG_07891	Ubiquitin-60S ribosomal protein L40	56,3684516880093
	**7. Transport routes**	
PADG_11111	NTF2 domain-containing protein	2,10378973395782
PADG_12077	Actin	2,69498705577783
PADG_00852	Aldolase_II domain-containing protein	*
	**8. Hypothetical protein**	
PADG_01692	Hypothetical protein	37,5551181102362
PADG_08212	Hypothetical protein	1,59222392377063
PADG_05703	Hypothetical protein	1,62044253998744
PADG_00140	Hypothetical protein	2,02050861361772
PADG_01857	Hypothetical protein	7,15993930197269

*Indicates that the protein is expressed specifically in the up-regulation condition

Table S2. ***P. brasilensis*** **proteins down-regulated after exposure to ZINCO8964784.**

Acession number	Protein description	Fold change
	**1. Metabolism**	
	**1.1 Amino acid metabolism**	
PADG_01404	Aspartate aminotransferase	0,612709226522582
PADG_01665	Aminotran_1_2 domain-containing protein	0,630313152400835
PADG_11902	Acetylornithine deacetylase	*
PADG_07029	Acetylornithine transaminase	*
PADG_05301	Cystathionine beta-synthase	*
PADG_01928	S-adenosylmethionine synthase	0,504704301075269
PADG_02719	Methionine adenosyltransferase 2 subunit beta	*
PADG_01566	Methylthioribulose-1-phosphate dehydratase	*
PADG_06252	1,2-dihydroxy-3-keto-5-methylthiopentene dioxygenase	*
PADG_08376	Aspartate-semialdehyde dehydrogenase	*
PADG_05277	Serine hydroxymethyltransferase	*
PADG_08304	Acetohydroxy-acid synthase small subunit	*
PADG_03167	3-isopropylmalate dehydratase	*
PADG_01718	Saccharopine dehydrogenase	0,389944775789706
PADG_01328	Ornithine aminotransferase	*
PADG_05058	Chorismate mutase	*
PADG_04522	Homoserine kinase	0,477443232969891
PADG_02777	Threonine synthase	*
PADG_07274	Multifunctional tryptophan biosynthesis protein	0,462079733527636
PADG_04854	Anthranilate synthase	*
PADG_06429	Ketol-acid reductoisomerase, mitochondrial	0,23989388812812
PADG_03020	Alanine-glyoxylate transaminase	*
PADG_12141	Clavaminate synthase	*
PADG_06319	Glutamate decarboxylase	*
PADG_00971	Glutamate decarboxylase	*
PADG_03686	Aspartate aminotransferase	*
PADG_03825	NAD-specific glutamate dehydrogenase	*
PADG_00405	Choline dehydrogenase	*
PADG_12025	Glutaryl-CoA dehydrogenase	0,38543066129273
PADG_06876	3-hydroxyisobutyryl-CoA hydrolase	0,282636293109528
PADG_01564	Methylmalonate-semialdehyde dehydrogenase	0,636197257251736
PADG_08464	Maleylacetoacetate isomerase	*
PADG_03964	FAA_hydrolase domain-containing protein	*
PADG_04142	FAA_hydrolase domain-containing protein	*
PADG_01032	Aminoacylproline aminopeptidase	*
	**1.2 Lipid, fatty acid and isoprenoid metabolism**	
PADG_11311	Glycerol kinase	*
PADG_00255	PKS_AT domain-containing protein	*
PADG_00254	Fatty acid synthase subunit alpha	*
PADG_06155	Palmitoyl-protein hydrolase 1	*
PADG_08651	Peroxisomal hydratase-dehydrogenase-epimerase	*

PADG_02751	Acetyl-CoA C-acetyltransferase	0,614730192049572
PADG_02244	(R)-benzylsuccinyl-CoA dehydrogenase	0,604582759412305
PADG_12125	Phytanoyl-CoA dioxygenase	*
	**1.3 C- compound metabolism**	
PADG_02145	Alpha-1,4 glucan phosphorylase	0,0453286197460781
PADG_07435	Sorbitol utilization protein SOU2	0,139206555057769
PADG_05778	Glycogen synthase	0,224937012948966
PADG_06165	GLTP domain-containing protein	0,465220165922144
PADG_12426	1,4-α glucan branching enzyme	*
PADG_00222	UDP-galactopyranose mutase	*
PADG_01677	Acetyl-coenzyme A synthetase	*
PADG_02733	D-lactate dehydrogenase	*
PADG_04374	UTP--glucose-1-phosphate uridylyltransferase	*
PADG_06731	Aldehyde reductase	*
	**1.4 Nucleotide metabolism and cycle cellular**	
PADG_00824	HABP4_PAI-RBP1 domain-containing protein	0,0704061470911087
PADG_01867	HMG box domain-containing protein	0,110961481334786
PADG_04312	UDP-N-acetylglucosamine pyrophosphorylase	0,150083257060309
PADG_05893	Histone chaperone NAP1	0,153988672968819
PADG_01488	S-adenosyl-L-methionine-dependent methyltransferase	0,390339474075175
PADG_04934	RNP domain protein	0,436574036998478
PADG_02683	UV excision repair protein RAD23	0,539720930232558
PADG_05474	Nudix hydrolase domain-containing protein	0,312850610474709
PADG_01100	Uracil phosphoribosyltransferase	0,645519326279421
PADG_07782	Deoxyuridine 5'-triphosphate nucleotidohydrolase	0,633967035713761
PADG_01100	Uracil phosphoribosyltransferase	0,645519326279421
PADG_00832	Adenylosuccinate synthetase	0,572224326552439
PADG_05683	Cell division control protein 48	0,513960800262792
PADG_00026	Cytidine deaminase	*
PADG_00604	Phosphoacetylglucosamine mutase	*
PADG_11857	Mitotic checkpoint protein BUB3	*
PADG_07670	SAP domain-containing protein	*
PADG_08483	Chromo domain-containing protein	*
	**1.5 Vitamins, cofactors and prosthetic groups**	
PADG_00607	Riboflavin synthase, α subunit	0,476773998944273
PADG_08480	RING-type domain-containing protein	0,115084887280824
PADG_04869	HIT domain-containing protein	0,46638824190828
PADG_03859	Protein BchC	0,645910765039269
	**1.6 Secondary metabolism**	
PADG_04687	3-β-hydroxysteroid dehydrogenase	0,133756418951652
PADG_03983	6,7-dimethyl-8-ribityllumazine synthase	0,446650011355894
PADG_03221	Mitochondrial intermediate peptidase	0,643905210551284
	**2. Energy**	
	**2. 1 Glycolysis and gluconeogenesis**	
PADG_03813	Hexokinase	0,185152910909166
PADG_07950	Hexokinase	0,329091922736366
PADG_00451	Glucose-6-phosphate isomerase	0,488844478145202

PADG_03118	Glucose-6-phosphate 1-epimerase	0,626097907868023
PADG_00192	ATP-dependent 6-phosphofructokinase	*
PADG_02132	Mannose-6-phosphate isomerase	*
PADG_01361	Triosephosphate isomerase	*
PADG_04103	Pyruvate carboxylase	0,307074355179608
PADG_00714	Pyruvate decarboxylase	0,551030891727181
	**2.2 Pentose-phosphate pathway**	
PADG_03651	6-phosphogluconate dehydrogenase	0,283805619419591
PADG_07606	D-xylose reductase	0,596690331608745
PADG_04604	Transketolase	0,621460780673835
PADG_07771	6-phosphogluconolactonase	0,56169953242529
	**2.3 Tricarboxylic-acid pathway**	
PADG_02063	Pyruvate dehydrogenase E1 component, alpha subunit	0,376318795779854
PADG_01762	α-ketoglutarate dehydrogenase E1 component	0,641457466924804
PADG_02805	Isocitrate dehydrogenase subunit, mitochondrial	*
PADG_03977	Isocitrate dehydrogenase subunit, mitochondrial	*
PADG_03058	Succinate-semialdehyde dehydrogenase	*
PADG_04994	ATP citrate synthase	*
	**2.4 Electron transport chain**	
PADG_00688	F-type H^+-transporting ATPase subunit H	0,281718664477285
PADG_11981	H^+-transporting two-sector ATPase	*
PADG_06956	Vacuolar proton pump subunit B	*
PADG_08394	Ubiquinol-cytochrome-c reductase subunit 2	0,195772768766238
PADG_07081	Electron transfer flavoprotein subunit alpha	*
PADG_11468	NADPH-dependent diflavin oxidoreductase 1	*
PADG_11513	NADH-ubiquinone oxidoreductase	*
PADG_04559	Cytochrome b5 heme-binding domain-containing protein	*
PADG_07813	ATP synthase subunit gamma	0,24766215039333
PADG_04729	ATP synthase subunit d, mitochondrial	*
PADG_11789	NAD(P)-bd_dom domain-containing protein	*
PADG_00366	NAD(P)-bd_dom domain-containing protein	0,368110387121502
	**3. Protein metabolism**	
PADG_03595	Xaa-Pro aminopeptidase	0,406301824212272
PADG_03965	Proteasome subunit beta	0,637760546347016
PADG_11319	Ubiquitin-conjugating enzyme variant MMS2	*
PADG_01051	Alpha-1,2-Mannosidase	*
PADG_02637	E2 ubiquitin-conjugating enzyme	*
PADG_04092	Peptidyl-prolyl cis-trans isomerase	0,205696711785005
PADG_01852	Hsc70 cochaperone	*
PADG_02761	Hsp75	*
PADG_02030	Hsp90	0,30587494273935
PADG_07715	Hsp90	0,426675376497635
PADG_04379	Hsp90 cochaperone STI1	0,644249053457212
PADG_04056	14-3-3	0,442636844174566
PADG_04440	14-3-3	0,4923549696451
PADG_12323	Peptidyl-prolyl cis-trans isomerase	0,485890481725429
PADG_02206	J domain-containing protein	*

PADG_04242	Sulfhydryl oxidase	*
PADG_08484	CCT-epsilon	*
PADG_00828	Peroxin-19	*
	**4. Cell rescue, defense and virulence**	
PADG_06740	Aldedh domain-containing protein	0,208358055773826
PADG_07699	S-formylglutathione hydrolase	0,245773468040818
PADG_02048	Nitroreductase domain-containing protein	0,350565428109855
PADG_03163	Cytochrome c peroxidase, mitochondrial	0,371533366499859
PADG_01486	Gluconate 5-dehydrogenase	0,408193243022686
PADG_07508	Phosphatidylinositol transfer protein SFH5	0,586649163577017
PADG_03161	Thioredoxin	*
PADG_03095	Thioredoxin	0,596719735656353
PADG_00324	Catalase	*
PADG_01626	Ferroxidase	*
PADG_11426	Cytochrome P450	*
PADG_03276	S-(hydroxymethyl)glutathione dehydrogenase	*
PADG_06273	Calcineurin regulatory subunit	*
PADG_06221	Formate dehydrogenase	*
PADG_11347	Cytotoxic granule associated RNA protein TIA1	*
	**5. Translation**	
PADG_00692	Elongation factor 1-alpha	0,511310514955288
PADG_01949	Elongation factor Tu	0,301383004236949
PADG_02896	EF1_GNE domain-containing protein	0,57469774398604
PADG_04057	Translation initiation factor 3 subunit J	0,320033042128714
PADG_06110	Translation machinery-associated protein 22	0,427457529375015
PADG_06249	Glutamine--tRNA ligase	0,454072951149336
PADG_01558	Histidine--tRNA ligase	0,329919143684326
PADG_07884	Polyadenylate-binding protein	0,446443683118585
	**6. Biogenesis of cellular components**	
PADG_07733	Arp2/3 complex 34 kDa subunit	*
PADG_08724	RPEL repeat protein	0,42800296956199
PADG_00128	Tubulin α-chain	*
PADG_05239	Tubulin-specific chaperone A	0,565254417713434
PADG_03959	Actin-related protein 2/3 complex subunit 1	*
PADG_07930	Actin-related protein 2/3 complex subunit 4	*
PADG_00945	Actin-related protein 2/3 complex subunit 5	*
PADG_08368	Actin-depolymerizing factor 1	*
	**7. Transport routes**	
PADG_06033	NIPSNAP family protein	0,092493833285102
PADG_03274	Mitochondrial import translocase subunit tim9	0,31901610687976
PADG_05884	Mitochondrial import translocase subunit tim10	0,329471182412359
PADG_05517	Rho GDP-dissociation inhibitor	0,381993569131833
PADG_02652	Grp1p	0,630514795921937
PADG_01243	Rab GDP dissociation inhibitor	0,520096744230932
PADG_00187	V-SNARE coiled-coil domain-containing protein	*
PADG_00282	GTP-binding protein ypt2	*
PADG_00442	Arf-GAP domain-containing protein	*
PADG_03475	ADP-ribosylation factor 6	*

PADG_02833	ADP-ribosylation factor	*
**8. Ribossomal metabolism**		
PADG_00784	40S ribosomal protein S0	0,48483292248699
PADG_06525	40S ribosomal protein S1	0,275149190110827
PADG_08602	40S ribosomal protein S2	0,658186428892797
PADG_01654	40S ribosomal protein S6	0,15691386784739
PADG_00942	40S ribosomal protein S7	0,347577683204104
PADG_07685	40S ribosomal protein S13	0,350470929820834
PADG_06313	40S ribosomal protein S18	0,519511967961255
PADG_06599	40S ribosomal protein S25	0,39712304135628
PADG_08605	40S ribosomal protein S28	0,416172186859424
PADG_11379	60S ribosomal protein L5	0,621062777898697
PADG_07803	60S ribosomal protein L12	0,422318438588777
PADG_03325	60S ribosomal protein L21	0,30275764797432
**9. Hypothetical protein**		
PADG_00211	Hypothetical protein	0,222384631447066
PADG_12256	Hypothetical protein	*
PADG_11657	Hypothetical protein	*
PADG_11480	Hypothetical protein	*
PADG_11588	Hypothetical protein	*

*Indicates that the protein is expressed specifically in the down-regulation condition
